# Interaction and main effects of physical and depressive symptoms on quality of life in Korean women seeking care for rectal prolapse: a cross-sectional observational study

**DOI:** 10.4069/kjwhn.2021.12.08

**Published:** 2021-12-20

**Authors:** Hee Moon, Youngrye Park, Mili Kim, Seonah Lee

**Affiliations:** 1 Department of Nursing, Sunchon National University, Sunchon, Korea; 2 Department of Nursing, Kunsan National University, Gunsan, Korea; 3 Chonnam National University Hospital, Gwangju, Korea; 4 College of Nursing, Chonnam National University, Gwangju, Korea

**Keywords:** Depressive symptoms, Physical symptoms, Quality of life, Rectal prolapse, Women

## Abstract

**Purpose:**

Rectal prolapse is still a relatively understudied medical condition, especially in women, whereas physical symptoms, depressive symptoms, and quality of life (QOL) in women with pelvic organ prolapse have been steadily studied. This study aimed to examine the interaction and main effects of physical and depressive symptoms on physical and mental QOL of women seeking care for rectal prolapse in Korea.

**Methods:**

Ninety-two women with rectal prolapse were recruited from a colorectal surgery clinic of a tertiary teaching hospital in Gwangju, Korea. Physical symptoms related to rectal prolapse (pelvic organ prolapse distress, POPD; colorectal-anal distress, CRAD; and urinary distress, UD), depression, and QOL were measured. The data were analyzed using descriptive statistics, Pearson correlation coefficient, and two-way analysis of variance.

**Results:**

The interaction between POPD symptoms and depressive symptoms (F=4.51, *p*=.037) affected physical QOL. The interaction between POPD (F=9.66, *p*=.003) and CRAD symptoms (F=7.48, *p*=.008), respectively, and depressive symptoms affected mental QOL. Depressive symptoms had a significant main effect on the physical QOL in the CRAD (F=6.22, *p*=.014) and UD (F=6.01, *p*=.016) groups and on mental QOL in the UD group (F=24.54, *p*<.001).

**Conclusion:**

Physical and depressive symptoms should be considered together to improve the QOL in women with rectal prolapse. Nursing interventions and medical treatments to decrease rectal prolapse-related physical and depressive symptoms are required to improve QOL in women with symptomatic rectal prolapse.

## Introduction

Rectal prolapse is a physically and emotionally distressing condition that can severely affect patients’ daily lives although it is not life-threatening [1]. The prevalence of rectal prolapse increases with advancing age, with a higher prevalence in women 50 years of age or over [1]. In South Korea (hereafter Korea), the prevalence rate of patients diagnosed with pelvic organ prolapse including rectal prolapse, increased by 3.76% from 2015 (2,362 patients) to 2019 (2,731 patients). Nearly 90% of the patients diagnosed with rectal prolapse were aged above 50 years, 34.7% were in their 70s, and 26.8% were in their 80s [2]. The relationship between pelvic floor disorders and age is thought to be due to aging changes in connective tissue integrity and neuromuscular degeneration [1,3]. Thus, age impacts the bothersome nature of rectal prolapse symptoms, especially as associated with functionality and quality of life (QOL) [1]. However, little is known about the prolapse-related physical and psychological problems and QOL in Korean women seeking care for rectal prolapse.

Rectal prolapse is a protrusion of part or all of the layers of the rectal wall toward or outside the anus [3]. It should not be confused with hemorrhoids, which are enlarged cushions of vascular tissue found within the anal canal in the submucosal space [4]. The underlying cause of rectal prolapse is unknown. However, known risk factors include lax muscles in the pelvic floor and anal canal, weak internal and external anal sphincters, diverse obstetric and gynecological surgeries, multiple vaginal deliveries, and straining [3,4]. Symptoms associated with rectal prolapse vary based on the anatomical defects or the degree of bowel dysfunction although some women have no symptoms despite the anatomical changes [5,6]. Defecatory symptoms are common [5], which include a protruding mass with bowel movements, mucous discharge, incomplete evacuation, constipation, fecal incontinence, and obstructed defecation [5,7]. These symptoms lead to pelvic floor dysfunction [3].

Prior studies have identified the myriad of negative effects stemming from pelvic organ prolapse, such as distress and embarrassment [6,8] and how women often suffer in silence [9], despite experiencing depressive symptoms [10] and low QOL [11,12]. Studies related to pelvic organ prolapse mainly reported relationships between prolapse-related physical symptoms and depressive symptoms and the main effects of these two groups of symptoms on the QOL of women with pelvic organ prolapse. However, limited studies provide an insight into the effects of physical and depressive symptoms on the QOL of women diagnosed with rectal prolapse. Specifically, there is insufficient evidence to suggest that the coexistence of physical symptoms and depressive symptoms worsens the QOL in women with rectal prolapse. This increases the need to investigate the interaction effects of physical symptoms and depressive symptoms on the QOL of women with rectal prolapse. An interaction effect refers to the combined effect of two or more independent variables on at least one dependent variable. The combined interaction effect of two or more independent variables is not equal to the sum of their separate effects, which indicates being able to be greater or less than the sum of their separate effects [13]. On the other hand, a main effect is the effect of an independent variable on a dependent variable averaged, ignoring the effects of other independent variables [13]. The interpretation of a main effect is straightforward if the interaction effect is not significant [14].

The purpose of this study was to examine the interaction and main effects of physical symptoms and depressive symptoms on the physical and mental QOL of women seeking care for rectal prolapse.

## Methods

Ethics statement: The study was approved by the Ethical Review Committee of Chonnam National University Hospital (CNUH-2016-308). Written informed consent was obtained from participants.

### Study design

This cross-sectional study used self-administered questionnaires via convenience sampling. This study was described in accordance with the STROBE (Strengthening the Reporting of Observational Studies in Epidemiology) guidelines (https://www.strobe-statement.org).

### Participants

Participants were recruited from the colorectal surgery clinic of a tertiary teaching hospital located in Gwangju, Korea. Eligibility criteria were women who were 50 years old or over, diagnosed with rectal prolapse, received nonsurgical treatment (e.g., monitoring disease status and pharmacotherapy), and understood the purpose of the study and the content of the questionnaire. Women who were scheduled for surgery due to severe symptoms of rectal prolapse were excluded, as surgical treatments would improve physical symptoms and QOL compared to women with nonsurgical treatments [15,16]. Women who had a history of any of the following were also excluded: colorectal or anal surgery with diagnoses other than rectal prolapse, radiotherapy for pelvic organs, neurologic disorders (e.g., spina bifida, multiple sclerosis, and stroke), and psychiatric disorders. Based on a significance level (α) of 0.05, an effect size of 0.4, and a statistical power of 0.95 using G*Power 3.1 software, the sample size required was a minimum of 84 participants [17]. Considering dropouts, 102 women were recruited and after excluding ten cases due to incompleteness, a total of 92 women participated in the study.

### Measurement

#### Physical symptoms

Physical symptoms related to rectal prolapse were measured using the three subscales of the Pelvic Floor Distress Inventory (PFDI)-20 developed by Barber et al. [18] and translated and modified by Yoo et al. [19] which measure symptoms experienced over the last 3 months: (1) the Pelvic Organ Prolapse Distress Inventory-6 (POPDI-6, six items) regarding bulging symptoms or heaviness in the pelvic area; (2) Colorectal-Anal Distress Inventory-8 (CRADI-8, eight items) regarding bowel problems; and (3) Urinary Distress Inventory-6 (UDI-6, six items) regarding urinary leakage or other lower urinary tract symptoms. The participant answers “yes” or “no” and then ranks the level of bother of each ‘yes’ item on a 4-point Likert scale (1, not at all to 4, quite a bit). Items answered with a “no” response receive a zero. The actual score is obtained by multiplying the mean of all of the responses with 25. Thus, each subscale score of the PFDI-20 ranges from 0 to 100. The higher the score, the greater the perceived level of distress. Cronbach’s alpha coefficient was from 0.70 to 0.93 for all three subscales [19] and 0.79 in this study. Cronbach’s alpha coefficients for pelvic organ prolapse distress (POPD), colorectal-anal distress (CRAD), and urinary distress (UD) were 0.60, 0.69, and 0.80, respectively, in this study.

#### Depression

Depression was measured using the seven items from the Hospital Anxiety and Depression Scale (HADS) translated by Oh et al. [20]. The HADS was developed for use in hospital settings but was also valid when being used in community settings [21]. Higher summed scores (range, 0–21) indicate greater perceived depression. A score of 0–7 is “normal,” 8–10 is “mild,” and 11–21 is “moderate to severe.” Cronbach’s alpha coefficient for depression items was 0.70 in community settings and 0.76 in clinical settings at development [22], 0.86 in a Korean sample [20], and 0.87 in this study.

#### Quality of life

QOL was measured using the Korean version [23] of the Short Form-12 Health Survey [24]. This 12-item instrument consists of two components: the physical component including physical functioning, role-physical (i.e., role limitations due to physical problems), bodily pain, and general health; and the mental component including vitality, social functioning, role-emotional (i.e., role limitations due to emotional problems), and mental health. Items are scored on a 2-point (1, yes and 2, no) or 6-point (1, all of the time to 6, none of the time) Likert, with the scores of physical and mental component summary transformed into a 0 to 100 range, respectively, according to the scoring manual using Quality Metric Health Outcomes scoring software 5.0 [23,25]. Higher scores indicate higher QOL. Cronbach’s alpha coefficients for the physical component summary and the mental component summary were 0.70 and 0.69, respectively, in this study.

#### Participants’ characteristics

With reference to previous studies [15,26,27], the characteristics of the participants included age, body mass index (BMI), parity, birth delivery methods, a history of hysterectomy, ever having used midlife hormone therapy, and medical conditions including allergic rhinitis and chronic obstructive pulmonary disease.

### Data collection

The data were collected at a colorectal surgery clinic between January 9 and August 25, 2017. When patients arrived at the waiting room and waited for their appointment, a research associate (MK) approached them and courteously asked them about participating in the study. For women who expressed interest, the researcher led them to the separate room and explained the study purpose, anonymity, and voluntary participation. After informed consent, participants filled out the questionnaires assisted by the researcher, when needed.

### Statistical methods

The IBM SPSS ver. 23.0 (IBM Corp., Armonk, NY, USA) was used for data analyses. Descriptive statistics were done for demographic data and the main variables. Correlations between physical symptoms, depressive symptoms, and physical and mental QOL were analyzed using Pearson's correlation coefficients. Participants were dichotomized into two groups depending upon the presence or absence of symptoms. The participants responding “somewhat,”, “moderately,”, or “quite a bit” to the POPDI and UDI were grouped as the POPD and the UD groups. Those who had no symptoms were considered the non-POPD and non-UD groups. As all participants experienced colorectal-anal symptoms, the participants scoring less than the median value of 37.50 were grouped as the low CRAD group and the remaining participants were considered the high CRAD group. The responses to depressive symptoms were also dichotomized as non-depressed (0 to 7 points) or depressed (≥8 points). The Kolmogorov-Smirnov test was used to test the normal distribution of the QOL data that was the dependent variable. The physical and mental QOL data were normally distributed as the *p*-values of the Kolmogorov-Smirnov test were .20 and .64, respectively, greater than .05. The total QOL data were also normally distributed with the *p*-value of .40 in the Kolmogorov-Smirnov test. Differences in QOL according to the characteristics of the participants were analyzed using the chi-squared test. The main effects and interaction effects of physical symptoms and depressive symptoms on the QOL were analyzed using two-way analysis of variance. Statistical significance was set at the level of the *p*-value less than .05 for all tests.

## Results

### Characteristics of the participants

The mean age of the 92 participants was 70.0±10.0 years old and 51.1% were overweight or obese (Table 1). The median parity value was 3 (range, 0-8), 70.7% of the participants (n=65) had more than three children, and 89.1% (n=82) experienced vaginal deliveries. Nearly 15% of the participants (n=14) answered “yes” to hysterectomy, 15.2% (n=14) to midlife hormone therapy, 18.5% (n=17) to allergic rhinitis, and 5.4% (n=5) to chronic obstructive pulmonary disease. There were no differences in QOL according to the characteristics of the participants.

### Physical symptoms, depressive symptoms, and quality of life perceived by the participants

The mean value for physical symptoms was 83.20 out of a total of 300 points (Table 2). In the subscales of the PFDI-20, the mean values in the POPD, CARD, and UD were 22.28, 38.27, and 22.19, respectively, out of a total of 100 points. The mean value for depressive symptoms was 7.83 out of a maximum of 21 points. Of 92 participants, 54.3% (n=50) reported the absence of depressive symptoms, 21.7% (n=20) had mild, and 23.9% (n=22) experienced moderate-to-severe depressive symptoms. The mean values of physical QOL and mental QOL were 38.06 and 42.50, respectively, out of a total of 100 points. The POPD, CRAD, UD, and depressive symptoms were positively correlated with each other, but negatively correlated with the physical and mental QOL. Physical QOL was positively correlated with mental QOL (Table 3).

### Differences in quality of life according to physical symptoms and depressive symptoms

QOL was examined according to the presence and absence of POPD symptoms and depressive symptoms (Table 4). The total mean values of the physical QOL were higher in the non-depression group (41.33 points) and the non-POPD group (40.56 points) than in the depression group (34.17 points) and the POPD group (37.54 points), respectively. The total mean values of both mental QOL were higher in the non-depression group (48.03 points) and the non-POPD group (46.36 points) than in the depression group (35.94 points) and the POPD group (41.69 points), respectively. The total mean values of the physical and mental QOL were higher in the low CRAD group and the non-UD group than in the high CRAD group and the UD group.

### Main effects of physical symptoms and depressive symptoms on quality of life 

Depressive symptoms had a significant main effect on physical QOL in the CRAD group (F=6.22, *p*=.014) and the UD group (F=6.01, *p*=.016). Depressive symptoms also had a main effect on mental QOL in all three groups: the POPD group (F=12.71, *p*=.001), the CRAD group (F=18.30, *p*<.001), and the UD group (F=24.54, *p*<.001). The POPD symptoms and CRAD symptoms had significant main effects on mental QOL (F=5.12, *p*=.026 and F=6.48, *p*=.013, respectively) (Table 5).

### Interaction effects of physical symptoms and depressive symptoms on quality of life

**Figure 1.**
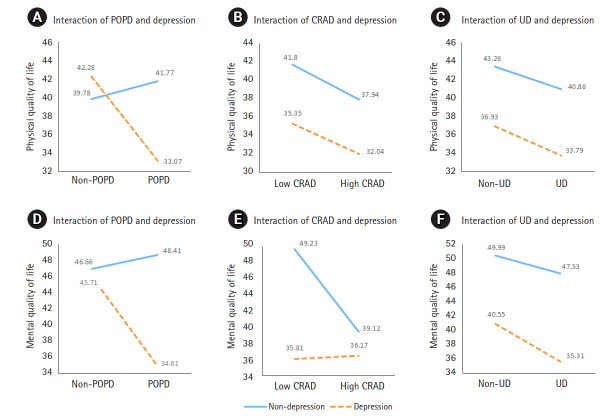
Main and interaction effects of physical symptoms and depressive symptoms on quality of life.(A, B, C) Interaction effects on physical quality of life. (D, E, F) Interaction effects on mental quality of life. CRAD: Colorectal-anal distress; POPD: pelvic organ prolapse distress; UD: urinary distress.

Presented as graphs to ease detection of interaction effects (lines not parallel or either intersecting, converging, or diverging) [28], the interaction between the POPD symptoms and depressive symptoms significantly affected both physical (F=4.51, *p*=.037) and mental QOL (F=9.66, *p*=.003) (Table 5; Figures 1A, 1D). The interaction between the CRAD symptoms and depressive symptoms significantly affected mental QOL (F=7.48, *p*=.008) (Table 5, Figure 1E). Thus, the non-parallel lines in Figures 1A, 1D, and 1E showed that there were interactive effects.

However, the interaction between the CRAD symptoms and depressive symptoms did not significantly affect physical QOL (F=0.01, *p*=.912) (Table 5, Figure 1C). The interaction between UD symptoms and depressive symptoms did not significantly affect physical QOL (F=0.02, *p*=.894) nor mental QOL (F=0.41, *p*=.526) (Table 5; Figures 1E, 1F). The parallel lines or non-crossing line in Figures 1B, 1C, and 1F show that there were no interaction effects.

## Discussion

This study analyzed the interaction and main effects of physical symptoms and depressive symptoms on the physical and mental QOL of women seeking care for rectal prolapse. There was an interaction effect between POPD symptoms and depressive symptoms on the physical QOL of women with rectal prolapse. There was also an interaction effect between POPD symptoms and CRAD symptoms, respectively, and depressive symptoms on the mental QOL. Therefore, POPD and CRAD symptoms and depressive symptoms should be simultaneously considered as factors affecting the physical or mental QOL of women seeking care for rectal prolapse. The results also showed that depressive symptoms had a significant main effect on the physical QOL of women in the CRAD and UD groups, and on the mental QOL of women in the UD group. In Figures 1B, 1C, and 1F, there were no interaction effects, and depressive symptoms had a true (statistically significant) main effect on the physical or mental QOL of women with rectal prolapse. This means that physical and mental QOL were higher in women without depressive symptoms than their depressed counterparts.

On the other hand, when the interaction is significant and crosses, it is not appropriate to try to interpret main effects because the main effects are not constant but vary according to the independent variables that interact with each other [14]. Usually, crossing lines mean no main effect [14]. In Figure 1-A, the lines crossed each other, indicating the interaction between the POPD symptoms and depressive symptoms. Therefore, it would be misleading to say that the POPD symptoms and depressive symptoms each had the independent main effect on physical QOL. In Figures 1D and 1E, although the lines did not intersect, there was the interaction effect between depression and POPD symptoms with CRAD symptoms, respectively, as well as with mental QOL. These interaction effects should be considered primary over the main effects of the POPD and CRAD symptoms on mental QOL.

To our knowledge, there has been no study that investigated the interaction and main effects related to physical symptoms, depressive symptoms, and QOL in women with rectal prolapse. However, as previous studies reported the relationships of depressive symptoms and physical symptoms on the QOL of women seeking care for diseases related to pelvic organ prolapse, the results of those studies were consistent with this study. In previous studies, pelvic organ prolapse symptoms were closely correlated with depressive symptoms [8,11,29]. Especially, depressive symptoms were highly prevalent among women with fecal and urinary incontinence and obstetric fistula attributable to pelvic organ prolapse [10]. Further, the physical symptoms attributable to prolapse were risk factors independently associated with the development of depressive symptoms in women with pelvic organ prolapse [8,29]. Women’s subjective feeling regarding not only the influence of prolapse itself on their QOL but also the impact of lower urinary tract symptoms and bowel dysfunction on their QOL were highly associated with depressive symptoms [8]. In the same vein, physical symptoms and depressive symptoms were independent risk factors contributing to the low QOL [11,12]. Pizarro-Berdichevsky et al. [11] reported that depressive symptoms, rather than anatomical abnormalities or objective clinical findings, determined how patients experience pelvic organ prolapse symptoms. Mazi et al. [29] suggested a bidirectional correlation between prolapse-related physical symptoms and depressive symptoms in women with pelvic organ prolapse as one of the two might represent the cause or effect on the other.

Based on the interaction of prolapse-related physical symptoms and depressive symptoms, it can be said that physical symptoms caused by rectal prolapse are more likely to induce depressive symptoms, and depressive symptoms also tend to exacerbate the physical symptoms due to rectal prolapse. Therefore, nursing interventions and medical treatments to decrease rectal prolapse-related physical symptoms and depressive symptoms at the same time are required to improve the QOL in women seeking care for symptomatic rectal prolapse. Further, screening for depressive symptoms should be implemented in the early stage of the diagnosis of rectal prolapse. The women who test positive for depressive symptoms need to be encouraged to obtain appropriate physical and psychological treatments.

This study had a few limitations. One is that the participants of this study were a convenience sample of women of 50 years of age or over diagnosed with rectal prolapse, recruited from a single colorectal surgery clinic. Therefore, the study findings cannot be generalized to all women diagnosed with rectal prolapse. Another is that cross-sectional nature does not allow causal inference. A longitudinal study design may be required to develop appropriate ways to address the physical symptoms, depressive symptoms, and QOL of women seeking non-surgical treatment for rectal prolapse, starting with diagnosis and during follow-up. Despite these limitations, however, this study had a strength as it examined the interaction effects of physical symptoms and depressive symptoms on the QOL of women seeking care for rectal prolapse. Further studies are needed to accumulate robust evidence supporting the interaction effects of physical and psychological symptoms on the QOL of women with rectal prolapse.

In conclusion, this study supports that physical symptoms related to rectal prolapse are closely correlated with depressive symptoms and their interaction has a significant influence on the QOL of women seeking care for rectal prolapse. Furthermore, depressive symptoms had a main effect on their QOL. Therefore, physical and depressive symptoms should be considered together to improve the QOL in women seeking care for rectal prolapse. Based on the findings, early identification of depressive symptoms and monitoring of physical symptoms are required, as are nursing interventions and medical treatments to decrease physical and depressive symptoms related to rectal prolapse.

## Figures and Tables

**Table 1.** Characteristics of the participants (N=92)

Variable	Categories	n (%)
Age (year)		Mean±SD, 70±10.0
	50–59	17 (18.5)
	60–69	26 (28.3)
	70–79	34 (37.0)
	≤80	15 (16.3)
Body mass index (kg/m^2^)	Underweight (≤18.5)	7 (7.6)
	Normal weight (18.6–22.9)	38 (41.3)
	Overweight (23.0–24.9)	21 (22.8)
	Obesity (≥ 25.0)	26 (28.3)
Parity		Median (range), 3 (0–8)
	0	10 (10.9)
	1–2	17 (18.5)
	≥ 3	65 (70.7)
Delivery methods	Vaginal delivery	82 (89.1)
	Cesarean section	10 (10.9)
Hysterectomy	Yes	14 (15.2)
	No	78 (84.8)
Midlife hormone therapy ever taken	Yes	14 (15.2)
	No	78 (84.8)
Allergic rhinitis	Yes	17 (18.5)
	No	75 (81.5)
Chronic obstructive pulmonary disease	Yes	5 (5.4)
	No	87 (94.6)

**Table 2.** Perceptions of physical symptoms, depressive symptoms, and quality of life (N=92)

Variable	Categories	Mean (SD)	Range	Possible range	Frequency (%)
Pelvic floor distress symptoms		83.20 (49.09)	6.25–233.33	0–300	
Pelvic organ prolapse distress			22.28 (19.81)	0–83.33	0–100	
Colorectal-anal distress		38.72 (18.88)	0–81.25	0–100	
Urinary distress		22.19 (23.16)	0–100	0–100	
Depressive symptoms		7.83 (4.67)	0–21	0–21	
	Normal	4.2 (2.10)	0–7	0–7	50 (54.3)
	Mild	8.95 (0.74)	8–10	8–10	20 (21.7)
	Moderate to Severe	14.36 (3.28)	11–21	11–21	22 (23.9)
Physical quality of life		38.06 (9.78)	20.11–57.29	0–100	
Mental quality of life		42.50 (9.54)	24.01–59.64	0–100	

**Table 3.** Correlations between physical symptoms, depressive symptoms, and quality of life (N=92)

Variable	Pelvic organ prolapse distress	Colorectal-anal distress	Urinary distress	Depressive symptoms	Physical quality of life
Pelvic organ prolapse distress	1				
Colorectal-anal distress	0.33**	1			
Urinary distress	0.54***	0.44***	1		
Depressive symptoms	0.48***	0.38***	0.37***	1	
Physical quality of life	–0.38***	–0.33**	–0.35**	–0.49***	1
Mental quality of life	–0.41***	–0.38***	–0.31**	–0.73***	0.48***

** *p*<.01,

*** *p*<.001.

**Table 4.** Differences in physical and mental quality of life according to the presence and absence of physical and depressive symptoms (N=92)

Group	Mean (SD)							
	Non-POPD	POPD	Low CRAD	High CRAD	Non-UD	UD	Non-depression	Depression
Physical quality of life	40.56 (7.95)	37.54 (0.09)	39.34 (9.91)	33.73 (8.14)	41.15 (9.53)	37.46 (9.78)	41.33 (8.99)	34.17 (9.34)
Mental quality of life	46.36 (6.18)	41.69 (9.95)	44.13 (9.36)	37.01 (8.14)	46.84 (8.37)	41.66 (9.57)	48.03 (7.59)	35.94 (7.19)

CRAD: Colorectal-anal distress; POPD: pelvic organ prolapse distress; UD: urinary distress.

**Table 5.** Main and interaction effects of physical symptoms and depressive symptoms on physical and mental quality of life (N=92)

Source	Physical QOL					Mental QOL				
	SS	df	MS	F	*p*	SS	df	MS	F	*p*
Model 1	1,579.43	3	526.48	6.50	.001	3,902.61	3	1,300.87	26.15	<001
POPD symptoms	151.29	1	151.29	1.87	.175	254.45	1	254.45	5.12	.026
Depressive symptoms	111.81	1	111.81	1.38	.243	632.06	1	632.06	12.71	.001
POPD × depressive symptoms	365.29	1	365.29	4.51	.037	480.28	1	480.28	9.66	.003
Error	7,130.92	88	81.03			4404.89	88	50.05		
Total	8,710.34	91				8,279.78	91			
Model 2	1,355.38	3	451.79	5.41	.002	3,874.89	3	1,291.63	25.80	<001
CRAD symptoms	174.62	1	174.62	2.09	.152	324.27	1	324.27	6.48	.013
Depressive symptoms	520.10	1	520.10	6.22	.014	915.74	1	915.74	18.30	<001
CRAD × depressive symptoms	1.03	1	1.03	.01	.912	374.27	1	374.27	7.48	.008
Error	7,354.96	88	83.58			4,825.07	88	54.83		
Total	8,710.34	91				8279.78	91			
Model 3	1,261.78	3	420.59	4.97	.003	3,503.4	3	1,167.80	21.52	<001
UD symptoms	87.48	1	87.48	1.03	.312	168.57	1	168.57	3.11	.081
Depressive symptoms	509.04	1	509.04	6.01	.016	1331.79	1	1,331.79	24.54	<001
UD × depressive symptoms	1.50	1	1.50	.02	.894	21.99	1	21.99	.41	.526
Error	7,448.56	88	84.64			4776.38	88	54.28		
Total	8,710.34	91				8,279.78	91			

CRAD: Colorectal-anal distress; MS, mean square; POPD: pelvic organ prolapse distress; QOL: quality of life; SS, sum of squares; UD: urinary distress.
